# Annotations

**Published:** 1907-05-25

**Authors:** 


### Annotations.

### Iron as a Constituent of Food.

Much discussion has taken place regarding the manner in which iron produces its therapeutic effects in the treatment of chlorosis. There are many difficulties in the way of believing that it is directly absorbed from the intestine, and, in addition, there is ground for the suggestion that the essential flaw in cases of chlorosis is not the absence of iron from the food supply, but an imperfect capacity for absorption by the blood-vessels of the intestinal mucous membrane. Based upon this latter contention is the view that inorganic iron cures chlorosis by improving the function of intestinal absorption and so permitting the inorganic iron present in various foodstuffs to reach the blood-stream. Whether this is the case or not, it is reasonable to remember that in the treatment of chlorosis such foods ought to have a prominent place. In any event they must materially contribute to the patient's progress. Dr. James J. Walsh has lately laid stress on this point, and he insists particularly on the value of red meats. Dr. Walsh claims that "the gravy from roast beef is nearly as effective as any iron preparation in the relief of the anæmia, and consequently also of the heart discomfort accompanying it." In addition, however, to red meat, many vegetables, it must be remembered, are able to contribute valuable proportions of iron to the dietary scheme. This is particularly true of the beet, yellow turnip, tomato, spinach, and green lettuce. These, therefore, are to be prescribed for chlorotic patients, and this not as mere occasional dietetic experiences, but daily, and even at several meals on each day. When this practice is adopted the practitioner need not be agitated by the controversy of organic *versus* inorganic iron. If with such a line of treatment he enforces abundance of rest and the cultivation of early hours, and prescribes so well established a remedy as Blaud's pills, he needs only perseverance to cure his cases of chlorosis. One other caution; let it be seen that the pills are freshly prepared and not the insoluble spheres which are turned out at a cheap price to cater for those who practise a false economy.

### Intubation of the Larynx in Diphtheria.

Dr. Claude B. Ker, of the Edinburgh City Hospital, has recently made an interesting contribution on the value of intubation in cases of laryngeal diphtheria. This method of treatment excited much interest when first introduced, but it hardly appears to have received anything like a general practical recognition. Dr. Ker now tells us that since 1894 it has been the operation of election at the hospital with which he is associated, and his conclusions are based on an experience of 200 cases. Until three years ago the mortality of the operation never fell below 40 per cent., a figure which is practically identical with the death rate in laryngeal cases at hospitals where tracheotomy is in vogue. In recent years, however, Dr. Ker can record a great improvement on these results, and he notes that this coincides with the systematic adoption of nasal feeding in all cases of intubation. He now depends, in these cases, solely and entirely on nasal feeding. Another modification which he has found desirable is the substitution of vulcanite for the old metal tubes. In hospital practice he is convinced that it is to the advantage of the patient to postpone operation as long as possible, and in most of his cases intubation was adopted only when the patient was becoming exhausted and the pulse was beginning to fail. This postponement gives the serum time to take effect, and it is quite safe when skilled assistance is continuously at hand. The conditions, however, are different in private practice, and here earlier operation is advisable, or even necessary. At the same time it is right to recognise that even with tolerably severe laryngeal paroxysms the patient may recover without operation. But this fact, though of great importance in hospital practice, is of little avail to the private practitioner. With definitely laryngeal symptoms the latter can hardly leave his patient without some interference, and hence tracheotomy is performed, and rightly, in cases which, in hospital, might be safely left alone. Dr. Ker's advice in these circumstances is to intubate as soon as laryngeal diphtheria is recognised, unless the obstruction is very marked, when tracheotomy is certainly the safer method.

### Waste in Convalescent Homes.

There can be no doubt that, as at present conducted, many convalescent homes fail to fulfil the purposes for which they are intended in the modern scheme of treatment of disease. Far too many cases are admitted who may not be physically ill. They consist of members of the community whose means are not large, who have run down in the course of the year's work, who will benefit by change and country air, and who obtain a letter from a convalescent home as the readiest and cheapest means of taking a rest in the country. The Convalescent Homes Association is making a valiant effort to bring the hospitals and convalescent homes more closely together, with the object of enabling an increasing number of beds at small additional expense to be made available for patients sent from a hospital who require more surgical attention than is generally afforded at present by convalescent homes. There is every reason why the managers of convalescent homes should welcome this movement, and why they should heartily co-operate to promote the supply of new surgical beds at existing convalescent homes. The movement has been checked by a lack in the supply of patients from the hospitals to the special surgical beds now available in convalescent homes. This, we understand, is due to a defect in the regulations which provide for the admission of such cases on one particular day in each week. To be effective for hospital purposes all such beds, when vacant, must be available for the receipt of patients on any day in the week. We are glad to hear that this difficulty will shortly be overcome, and that there is every prospect that the work of the Convalescent Homes Association will steadily develop to the utmost the inter-relations which ought to exist between hospitals and convalescent institutions.

